# Large Language Models for Drug-Related Adverse Events in Oncology Pharmacy: Detection, Grading, and Actioning

**DOI:** 10.3390/pharmacy13060176

**Published:** 2025-12-03

**Authors:** Md Muntasir Zitu, Ashish Manne, Yuxi Zhu, Wasimul Bari Rahat, Samar Binkheder

**Affiliations:** 1 Department of Machine Learning, Moffitt Cancer Center and Research Institute, Tampa, FL 33612, USA; 2 Department of Medicine, College of Medicine, The Ohio State University, Columbus, OH 43210, USA; 3 Department of Pediatrics, University Hospitals Rainbow Babies & Children’s Hospital, Cleveland, OH 44106, USA; 4 Department of Computer Science and Engineering, East West University, Aftabnagar, Dhaka 1212, Bangladesh; 5 Medical Informatics and E-Learning Unit, Medical Education Department, College of Medicine, King Saud University, Riyadh 12372, Saudi Arabia

**Keywords:** large language model, pharmacy, adverse events, oncology, artificial intelligence, natural language processing, clinical texts

## Abstract

Preventable medication harm in oncology is often driven by drug-related adverse events (AEs) that trigger order changes such as holds, dose reductions, delays, rechallenges, and enhanced monitoring. Much of the evidence needed to make these decisions lives in unstructured clinical texts, where large language models (LLMs), a type of artificial intelligence (AI), now offer extraction and reasoning capabilities. In this narrative review, we synthesize empirical studies evaluating LLMs and related NLP systems applied to clinical text for oncology AEs, focusing on three decision-linked tasks: (i) AE detection from clinical documentation, (ii) Common Terminology Criteria for Adverse Events (CTCAE) grade assignment, and (iii) grade-aligned actions. We also consider how these findings can inform pharmacist-facing recommendations for order-level safety. We conducted a narrative review of English-language studies indexed in PubMed, Ovid MEDLINE, and Embase. Eligible studies used LLMs on clinical narratives and/or authoritative guidance as model inputs or reference standards; non-text modalities and non-empirical articles were excluded. Nineteen studies met inclusion criteria. LLMs showed the potential to detect oncology AEs from routine notes and often outperformed diagnosis codes for surveillance and cohort construction. CTCAE grading was feasible but less stable than detection; performance improved when outputs were constrained to CTCAE terms/grades, temporally anchored, and aggregated at the patient level. Direct evaluation of grade-aligned actions was uncommon; most studies reported proxies (e.g., steroid initiation or drug discontinuation) rather than formal grade-to-action correctness. While prospective, real-world impact reporting remained sparse, several studies quantified scale advantages and time savings, supporting an initial role as high-recall triage with pharmacist adjudication. Overall, the evidence supports near-term, pharmacist-in-the-loop use of AI for AE surveillance and review, with CTCAE-structured, citation-backed outputs delivered into the pharmacist’s electronic health record order-verification workspace as reviewable artifacts. Future work must standardize reporting and CTCAE/version usage, and measure grade-to-action correctness prospectively, to advance toward order-level decision support.

## 1. Introduction

Preventable medication harm remains a central safety challenge in oncology, where polypharmacy, narrow therapeutic indices, organ dysfunction, and evolving protocols increase the risk of error [1,2,3]. Oncology pharmacists are expected to function as medication-safety leaders and the safeguard before order execution, with explicit responsibilities for independent verification of doses, routes, schedules, and system-level error prevention [1,4].

Within this landscape, drug-related adverse events (AEs) drive a large share of clinically important order changes (hold, dose reduction, delay, rechallenge, enhanced monitoring). AEs are common in cancer populations. Systematic reviews and cohort studies report high rates of treatment-related toxicities, particularly with oral oncolytics and supportive therapies, underscoring the need for proactive pharmacist triage [2]. On the toxicity side, oncology standardizes severity using the Common Terminology Criteria for Adverse Events (CTCAE), which maps signs, symptoms, and laboratory abnormalities to graded levels that trigger specific clinical actions [5]. Newer modalities add domain-specific frameworks: immune effector cell therapies require consensus grading for cytokine release syndrome (CRS) and immune effector cell–associated neurotoxicity syndrome (ICANS) from the American Society for Transplantation and Cellular Therapy, while immune checkpoint inhibitor (ICI) care relies on guideline-based management of immune-related AEs (irAEs) [6,7,8,9,10].

These safety decisions rely predominantly on text, such as clinic and infusion notes, telephone encounters, discharge summaries, and narrative reports. Manually processing these narratives at scale, however, is inherently difficult [11]. Multiple overviews estimate that a large proportion of clinically relevant EHR information is unstructured and resides in narrative text, motivating text-centric methods for surveillance and decision support [12]. In oncology specifically, investigators have shown that Artificial Intelligence (AI)-based automation, such as Natural Language Processing (NLP) techniques, could identify irAEs from clinical narratives and improve mapping to structured labels, demonstrating the feasibility of AE surveillance [13,14,15].

**Figure 1:**
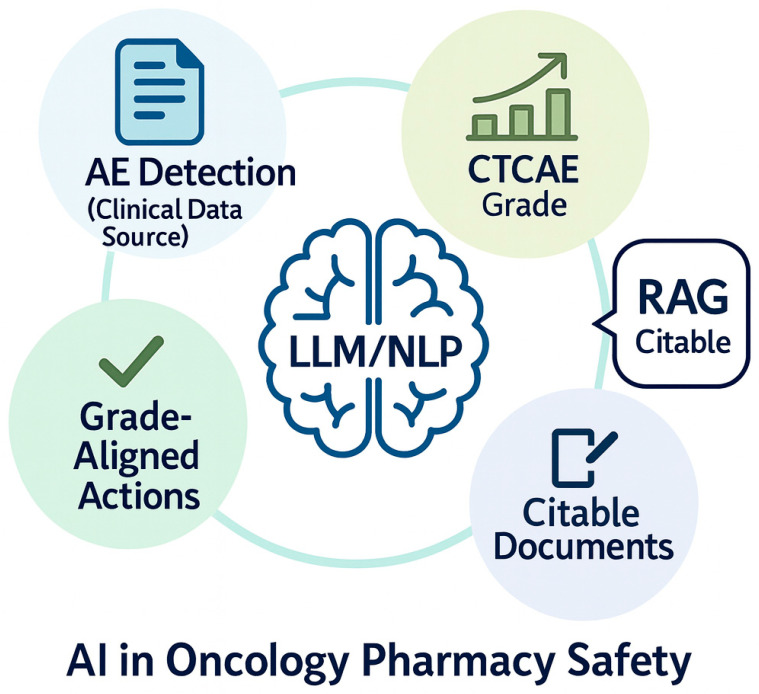
Illustration of the role of large language models (LLMs) and natural language processing (NLP) in enhancing oncology pharmacy safety. The figure depicts three interrelated domains: (1) Adverse Event (AE) detection from clinical notes, enabling early identification of safety risks; (2) CTCAE grade assignment, mapping toxicity severity using standardized criteria; and (3) Grade-aligned actions such as holding, restarting, dose-adjusting, or monitoring treatment based on toxicity grading, supported by retrieval-augmented generation (RAG) to surface citable evidence from trusted guidelines and references in real time.

Over the last several years, more advanced AI approaches such as large language models (LLMs) have expanded what is possible for extraction and reasoning from clinical text, but they are fallible in high-stakes settings without safeguards [16,17,18,19]. LLMs can produce factually incorrect or unfaithful statements (“hallucinations”) and omissions, reinforcing the need for designs that foreground provenance and constrain outputs [20]. One consistently supported mitigation is retrieval-augmented generation (RAG), which conditions generation on retrieved, citable documents and has been shown to improve factual specificity on knowledge-intensive tasks [21]. Global public-health bodies likewise urge caution and governance when deploying LLMs in healthcare workflows [22]. Figure 1 illustrates an AI (LLM/NLP) workflow oriented to oncology pharmacy, emphasizing AE detection from clinical text, CTCAE grade assignment, and grade-aligned actions supported by retrieval and citation.

Our narrative review focuses on oncology-pharmacy safety use cases where LLMs could support verification and propose candidate actions at medication order review: (i) AE detection from clinical documentation, (ii) CTCAE grade assignment, and (iii) grade-aligned actions (hold/reduce/delay/rechallenge/monitor). These three focal tasks in this review directly align with established oncology pharmacy responsibilities and safety checks. The American Society of Clinical Oncology/Oncology Nursing Society (ASCO–ONS) (ASCO–ONS) antineoplastic therapy administration standards require independent verification, documentation, and escalation processes that place pharmacists at the safety gate for order review and changes, across settings and routes of administration [1,23]. Professional guidance from Hematology/Oncology Pharmacy Association HOPA likewise defines pharmacists’ core roles in therapy selection, ongoing medication management, and monitoring/management of adverse effects, underscoring pharmacist leadership in toxicity surveillance and actioning [24]. Empirical safety work shows that pharmacist-designed verification tools embedded in computerized provider order entry (CPOE), such as an electronic chemotherapy order-verification (ECOV) checklist fit naturally into sequential pharmacist evaluation. These tools have been shown to increase capture of reportable medication errors (i.e., “good catches”), reinforcing the value of structured, review-ready outputs for adjudication [25]. Historical and contemporary evidence further demonstrates that oncology medication errors are common and clinically significant without robust verification, motivating pharmacist-centered surveillance and decision support [26,27,28]. Finally, the CTCAE framework (and American Society for Transplantation and Cellular Therapy or ASTCT grading for cellular therapies) is the prevailing standard for toxicity severity and management, justifying our emphasis on CTCAE-aligned, provenance-backed outputs rather than unconstrained free-text [6,29].

In this narrative review, we synthesize empirical studies from 2018 onward that utilize LLM techniques on text data in oncology settings. Our review comprises retrospective analyses, prospective vignette/simulation work, and prospective real-world deployments. Our objective is to describe how these systems are being used for oncology-pharmacy safety decisions (AE detection, CTCAE grading, and grade-aligned actions), to assess the strength and limits of the reported evidence, and to clarify the data and design conditions under which they appear reliable enough for pharmacist-in-the-loop use. In doing so, we make three contributions: we consolidate what is known about model performance across detection, grading, and actioning; we identify practice-relevant gaps (especially limited reporting of grade-to-action correctness and prospective impact); and we translate the evidence into pharmacist-centered recommendations and integration patterns aimed at order-level safety. Please refer to Table 1 for the acronyms and the Glossary of terms to better understand our study.

## 2. Methodology

### 2.1. Scope of Review (Inclusion and Exclusion)

**Figure 2:**
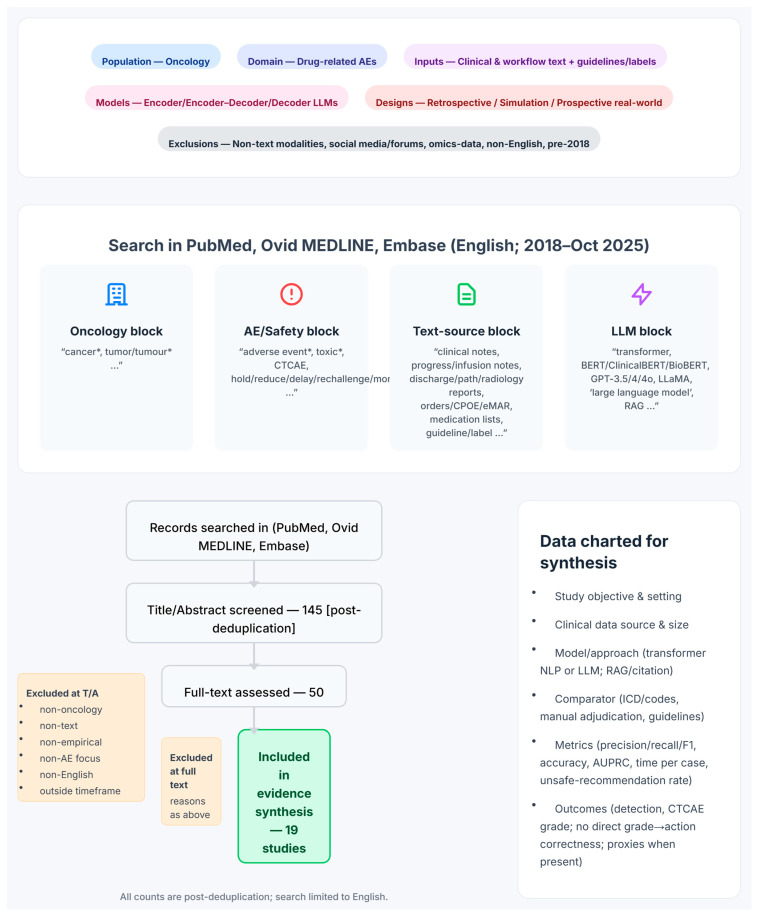
Methodology for our narrative review. The infographic provides a visual summary of the search strategy, study selection process, and data extraction protocol. A systematic search of PubMed, Ovid MEDLINE, and Embase (from January 2018 to October 2025) was conducted using queries constructed from four conceptual blocks (oncology, AE/safety, text source, and LLM terms). The study selection funnel illustrates the screening process, starting from 145 records assessed at the title/abstract level down to the 19 studies included in the final evidence synthesis. The key data points charted from each study are outlined, and the review’s overall scope and exclusion criteria are defined.

This narrative review focuses on oncology-pharmacy safety decisions for the oncology patient population, limited to one domain: drug-related AEs. In contrast to systematic reviews, which employ stringent inclusion criteria and aim to comprehensively encompass all relevant literature, our narrative review utilizes a more flexible and interpretive approach [30,31]. We include empirical studies that applied LLMs (encoder-only transformers, encoder–decoder models, and decoder-only/generative LLMs) as well as techniques such as citation-enforced RAG [21,32]. Studies that utilized only NLP methods without incorporating transformers/LLMs were excluded from the analysis. The included studies focus on various aspects of AE extraction, ranging from methodology development for AE detection to CTCAE grade assignment or grade-aligned actions (hold, reduce, delay, rechallenge, or monitor). Included inputs for the LLMs comprise clinical narratives and workflow text (physician/clinic/infusion notes, telephone encounters, discharge summaries, pathology/radiology reports, orders/CPOE/electronic medication administration record or eMAR, medication lists) as well as authoritative reference text such as guidelines or labels. Non-text modalities (e.g., imaging, waveforms, omics) along with social media/forums and bulk pharmacovigilance narratives are excluded from this study. We include empirical evaluations, which encompass retrospective analyses, prospective vignette/simulation studies, and prospective real-world deployments. Our review includes only articles written in the English language from 2018 to October 2025 (the transformer/LLM era), encompassing peer-reviewed articles, abstracts, and preprints [32,33]. The methodology for this review, including the search strategy, study selection process, and data extraction protocol, is visually summarized in Figure 2.

### 2.2. Article Selection, Literature Search, and Review

We performed a structured search of PubMed, Ovid MEDLINE, and Embase (2018–October 2025) using four Boolean blocks combined with AND and OR in the Title/Abstract section. Those four Boolean blocks included an oncology block (e.g., cancer, tumor), a safety block focused on adverse events (e.g., adverse event, toxicity, CTCAE), a text-source block (e.g., clinical notes, discharge/pathology/radiology reports, authoritative guidelines/labels), and an LLM block (transformer/LLM terms spanning encoder-only, encoder–decoder, generative models and RAG, with common model names and synonyms). A typical Title/Abstract Boolean structure was: (cancer OR oncology OR malignant) AND (“large language model*” OR LLM* OR “language model*” OR “generative AI” OR “generative model*” OR “GPT-4” OR “GPT-3” OR ChatGPT OR BERT OR Longformer OR LLaMA OR “Med-PaLM” OR Gemini OR Claude OR RAG OR “retrieval-augmented”) AND (guideline* OR NCCN OR ASCO OR ESMO OR “tumor board” OR “electronic health record*” OR EHR OR “clinical note*” OR “discharge summar*” OR “progress note*” OR “triage note*” OR “pathology report*” OR “radiology report*”) AND (“adverse event*” OR toxicity OR toxicities OR AE OR AEs OR CTCAE OR “Common Terminology Criteria”). All three databases were last accessed on 10 October 2025. Searches were limited to articles that were published in English (However, the data analyzed in those articles may originate from other languages, such as Japanese.); duplicates across databases were removed. Two reviewers independently screened titles/abstracts and, for potentially eligible records, full-text articles and extracted data using a structured form, resolving disagreements by discussion. This yielded 145 records based on title/abstract for manual review. Fifty records passing this stage underwent full-text manual review for final selection. The final evidence set comprises 19 studies, which serve as the sole basis for synthesis and discussion in this review.

## 3. Results

### Overview of Selected Articles

**Figure 3:**
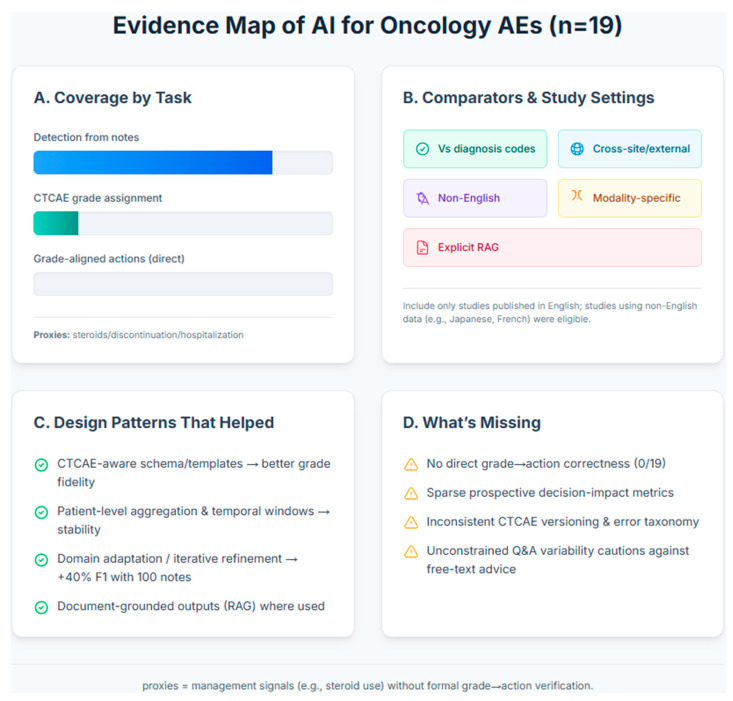
Evidence Map of AI for AE assessment in oncology. This infographic summarizes the landscape of 19 studies on AI-based assessment of oncology AEs from text data. The evidence shows a strong focus on AE detection from clinical notes, with significantly less attention on assigning CTCAE grades Panel (**A**). The research landscape is characterized by limited cross-site validation and minimal use of explicit RAG techniques Panel (**B**). Key successful design patterns include using CTCAE-aware schemas for better grade fidelity and patient-level data aggregation for stability Panel (**C**). Major gaps remain in validating grade-to-action correctness, measuring prospective decision impact, and standardizing error taxonomies across studies Panel (**D**).

The final evidence set comprises 19 empirical studies (Table 2). These studies are oncology-focused and evaluate LLMs on text data for drug-related AEs. Across the set, most studies focused on AE detection from clinical narratives (clinic/infusion progress notes, follow-ups, often with discharge, pathology, or radiology reports), while a smaller subset evaluated CTCAE grade assignment. No study directly evaluated grade-aligned actions against CTCAE or local policy. However, a few studies reported proximal management signals (e.g., steroid initiation) rather than verifying hold/reduce/delay/rechallenge/monitor. Inputs were predominantly EHR narratives, with several studies using CTCAE/guideline excerpts or expert adjudication as the reference standard; some systems incorporated citation-oriented retrieval, CTCAE-aware prompting, and structured output schemas. Model approaches spanned encoder transformers (e.g., BERT-family classifiers) and generative LLMs, with study designs ranging from retrospective cohorts to prospective vignette/simulation and limited prospective real-world evaluations. Reported endpoints centered on precision/recall/F1 for detection and accuracy for grading; decision-impact metrics (e.g., time per case, acceptance/override, unsafe-recommendation rate) were inconsistently reported, and grade-to-action correctness was not measured. Figure 3 shows the evidence map summary.

## 4. Discussion

### 4.1. Synthesis of the Selected Articles

#### 4.1.1. What’s Most Mature: AE Detection from Clinical Narratives

Across the evidence set, the most consistent signal is that transformer/LLM pipelines can identify oncology adverse events from routine clinical text with usable accuracy, often exceeding diagnosis codes for surveillance or cohort-building in multiple cohorts. Multi-institutional work with GPT-3.5/4/4o achieved patient-level micro-F1 ≈ 0.56–0.59 across two centers (VUMC, UCSF), with organ-category micro-F1 ≈ 0.61–0.66 after simple aggregation, demonstrating evidence of cross-site generalizability for irAE phenotyping from notes [36]. LLM-assisted “augmented curation” outperformed diagnosis codes for immune-related AEs and captured steroid use as an action proxy (F1 ≈ 0.84; steroid use ≈82%) [35]. In a broader irAE pipeline comparison, the LLM approach reported higher sensitivity/PPV/NPV than ICD coding and compressed chart-review time from ~9 weeks to ~10 min for ~9000 patients, underscoring scalability for safety surveillance [51]. A separate prospective comparison versus ICD demonstrated a mean sensitivity of ≈95% (specificity ≈ 94%) at the encounter level but low PPV (~15%), arguing that LLM outputs are best used as triage cues rather than final labels [52].

In practical terms, these AE-detection systems all tackle a similar problem: turning free-text oncology documentation into patient- or encounter-level flags that one or more CTCAE-defined toxicities are present in a clinically relevant time window around treatment. Most pipelines utilize oncology progress notes, discharge summaries, radiology or nursing documentation, and use LLM-based classification or extraction to map those narratives to binary or multi-label AE indicators, sometimes with simple rules layered on top. Reference standards are typically manual chart review or curated irAE/toxicity cohorts, and several groups explicitly compare against diagnosis-code–only baselines, showing that note-based models consistently recover hidden true events at acceptable precision. This convergence across immune-checkpoint inhibitors, radiotherapy toxicities, and broader oncology AEs is what makes detection from clinical narratives the most empirically mature use case in this literature, even though downstream grading and treatment-action recommendations still require human oversight.

Collectively, these results support a “detect-to-triage-to-adjudicate” role for LLMs in oncology pharmacovigilance. High recall surfaces candidate cases, while pharmacists (or trained abstractors) adjudicate details, an operational pattern that several groups explicitly highlight in their “Clinical Impact/Advancements” statements [35,36,51,52].

#### 4.1.2. From Detection to Grading: CTCAE Alignment Is Feasible but Harder

Moving from “did an AE occur?” to “how bad is it?” consistently reduces performance, yet multiple studies show that CTCAE-aware templates or task-specific training make the step tractable. In radiation-oncology notes and reports, a classifier achieved macro-F1 ≈ 0.92/0.82/0.73 across progressively more challenging esophagitis grading tasks (note-, series-, patient-level), providing one of the clearest demonstrations of automatic CTCAE severity extraction from routine text [39]. A GPT-4–based classifier obtained ~82–86% accuracy for general chemotherapy-related toxicity classes, but performance fell for fine-grained grades (0–4), a pattern consistent with the narrative that coarse category mapping is easier than exact grade calls [38]. Predictive modeling over longitudinal oncology notes also reached macro-AUPRC ≈ 0.58 for common symptomatic toxicities (nausea/vomiting, fatigue/malaise), outperforming an open-source baseline and suggesting value for early-warning workflows that feed pharmacist review [48]. Together, these studies indicate that grade mapping is feasible, but reliability depends on anchoring to CTCAE language, careful time-windowing, and (often) specialty-specific corpora [38,39,48].

#### 4.1.3. Toward Grade-Aligned Actions: Early Signals, Limited Direct Evaluation

Far fewer studies evaluate actions (hold/reduce/delay/rechallenge/monitor) directly, but several provide proximal evidence. An augmented-curation pipeline quantified steroid initiation for irAEs, linking detection to a management action [35]. A large irAE cohort reported real-world management actions (e.g., steroid percentages) alongside detection metrics, illustrating how LLM pipelines can instrument downstream care [51]. Another prospective comparison highlighted that, although recall was high, precision was modest, reinforcing the need for structured, review-ready outputs (e.g., explicit grade and action candidates with provenance) before order changes are made [52]. In sum, action correctness is under-reported across the literature; most studies stop at detection or grade, with only a few quantifying grade-to-action or using action proxies (steroids). This gap motivates the recommendations we present later.

#### 4.1.4. Modality-Specific Toxicity Use Cases (CAR-T, Radiotherapy, Antibody–Drug Conjugates)

LLM-based systems are already probing modality-specific safety needs. In cellular therapy, GPT-4 extracted CAR-T–related AEs with ~64% manual-validation accuracy and identified clusters (e.g., encephalopathy/neurologic) at a ~13-day post-infusion mean, promising for post-CAR-T monitoring despite remaining headroom [43]. For late radiation toxicities, a teacher–student LLM pipeline reached overall accuracies ~84% after per-symptom refinement, supporting longitudinal survivorship surveillance [42]. In antibody–drug conjugate safety (trastuzumab deruxtecan), a BERT-based note classifier plus clinical review surfaced interstitial lung disease events (n = 16) and linked them to management outcomes, illustrating combined algorithmic and clinician workflows for high-risk drug AEs [40].

#### 4.1.5. Non-English and Cross-System Generalizability

Several studies demonstrate feasibility beyond English and across disparate care settings. Japanese pharmacy and hospital notes supported ADE NER and normalization with patient-level precision ≈0.88, recall 1.00 (F1 ≈ 0.93) for a capecitabine-induced hand–foot syndrome use case, and broader symptomatic AE detection with exact-match F1 ≈ 0.72–0.86 [44,46]. Two large studies emphasized cross-site performance: ClinicalBERT transferred between institutional corpora with F-score ~0.74–0.78 and achieved ~0.87 within-dataset [50], while organ-category irAE performance replicated across VUMC/UCSF cohorts [36]. Notably, where the text comes from matters, detectability of certain AEs varied by note source (e.g., oncology vs. pharmacy notes), a pragmatic insight for deployment planning [46,47].

Overall, the studies above, particularly on non-English datasets, are valuable stress tests, but they also highlight limits on generalizability. Models trained on a single language and site-specific documentation style (e.g., templated radiology reports, local abbreviations, or institution-specific note structures) may overfit to those patterns and perform less well when applied to English notes, different EHR vendors, or community settings. Such shifts can introduce hidden bias in adverse event detection if certain toxicities are described differently or under-documented in new environments. Future work should therefore report cross-language and cross-system validation, or explicitly use adaptation (e.g., translation, local fine-tuning) before deploying these systems for safety-critical oncology pharmacy decisions.

#### 4.1.6. Guardrails and Design Patterns That Helped

Across studies, three patterns recur when performance and usability improved:

(a) Task-specific schemas or CTCAE-aware prompting. Making the model “speak CTCAE” via templates or label spaces improved grade fidelity and interpretability [38,39].
(b) Patient-level aggregation and temporal anchoring. Aggregating sentence- or note-level signals to the patient timeline (and constraining to clinically plausible windows) stabilized performance across sites [36].
(c) Retrieval/citation and domain adaptation. Systems that incorporated citation-style retrieval or modest domain-adaptation (e.g., small in-domain labels) reported sizeable gains, for instance, +40% F1 with 100 annotated notes versus zero-shot [44] and improved precision for post-RT symptoms after per-symptom refinement [42].

By contrast, generic Q&A without constraints showed omissions and variability for safety content: GPT-4 answered AE questions correctly only ~53% of the time, with 76% answer variability on repeats, underscoring the need for structured outputs, retrieval, and verification in clinical safety contexts [45]. Even in stronger pipelines, recall-heavy behavior can depress PPV (e.g., PPV ≈ 15% in a high-sensitivity irAE screen), again arguing for pharmacist triage before action [52].

#### 4.1.7. Scale, Workload, and “Fit for Use”

Several groups quantified workflow impact or scale: one pipeline processed ~9000 patients in ~10 min, turning an otherwise infeasible manual review into a tractable queue for human adjudication [51]. Others framed their contribution as registry or real-world evidence enablement. For example, studies demonstrate capturing post-treatment symptom recurrence and complications with recall ~100% (local recurrence) and F1 ≈ 0.80 (pneumothorax) for key events [41], or prospective monitoring where early-warning predictions can focus team attention [43,48]. Importantly, multiple studies emphasize that the intended use matters: systems with high recall and explainable outputs are “fit” for surveillance and triage, whereas grade-to-action automation requires CTCAE-anchored structure, tighter precision, and explicit provenance [35,39,52].

#### 4.1.8. What Remains Under-Reported

Two gaps cut across the literature. First, grade-aligned action correctness is measured infrequently; steroids serve as a proxy in a few irAE cohorts, but comprehensive evaluation of hold/reduce/delay/rechallenge/monitor recommendations is rare [35,51]. Second, unsafe-recommendation taxonomies and versioning of CTCAE/guidelines are inconsistently reported, limiting reproducibility and auditability [multiple studies’ “Clinical Impact/Advancements” notes]. Addressing these will be central to moving from surveillance to order-level decision support.

## 5. Future Directions

### 5.1. Start with Surveillance and Triage

**Figure 4:**
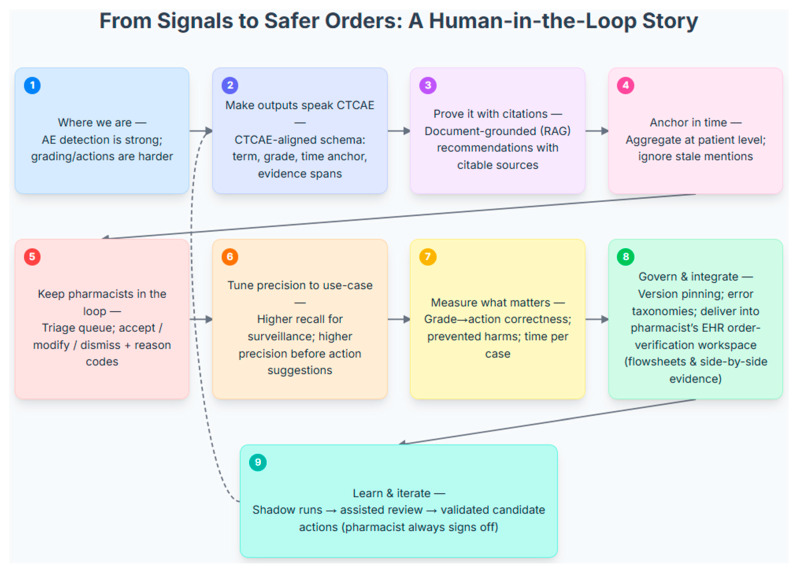
A Human-in-the-Loop Workflow for Processing Adverse Event Signals into Actionable Clinical Recommendations. This figure illustrates a nine-step, human-in-the-loop workflow that translates AE signals into safe, pharmacist-verified clinical actions. The process begins by structuring raw AE data into a clinically relevant format by aligning it with a CTCAE schema, grounding recommendations in citable sources (RAG), and aggregating timely patient-level data (Steps 1–4). A pharmacist provides essential oversight by reviewing, modifying, or dismissing all system-generated suggestions in a dedicated triage queue (Step 5). The system’s precision is tuned to the clinical use-case, and its performance is measured by the correctness of grade-to-action mapping, prevented harms, and time per case (Steps 6–7). Designed for robust deployment, the model integrates into the pharmacist’s EHR workspace (Step 8) and is underpinned by an iterative learning model. A continuous feedback loop (Step 9 to 2) allows the system to learn from pharmacist decisions, while the clinician always retains final sign-off authority.

Consistent with the scope defined in the Introduction, the most reliable use of LLMs today is AE detection from routine oncology narratives, with more variable performance for CTCAE grading and limited evaluation of grade-aligned actions. Accordingly, initial deployment should position the model as a surveillance/triage service that sweeps clinic, infusion, and follow-up notes to surface high-recall AE candidates. Outputs should be delivered into the pharmacist’s EHR order-verification workspace as review items rather than as order-changing directives, leveraging sensitivity without compromising safety or accountability. Figure 4 illustrates various aspects of future directions and recommendations, as well as their interconnections from signals to safer orders with a human-in-loop.

### 5.2. Make Outputs Speak CTCAE

For efficient review and documentation, model outputs should follow a CTCAE-aligned schema: toxicity term, proposed grade, temporal anchor (onset/resolution), and the specific evidence span(s) supporting the call. Constraining results to this structure converts free-text claims into reviewable objects, reduces ambiguity, and shortens the path from “possible AE” to a pharmacist determination of actionability during order-verification.

### 5.3. Prove Recommendations with Citations

Whenever grade-aligned suggestions are offered (hold, reduce, delay, rechallenge, monitor), recommendations should be document-grounded. Retrieval-augmented prompts should display the exact supporting note excerpt. Additionally, where relevant, they should show the corresponding CTCAE passage or versioned local policy (with date/source), providing the provenance required for clinical trust. The model assembles and cites the evidence; pharmacists retain judgment and final sign-off.

### 5.4. Anchor in Time and at the Patient Level

AE interpretation is inherently temporal. Systems should aggregate sentence- and note-level signals to the patient level, suppress stale/problem-list mentions, and constrain evidence to clinically plausible windows (e.g., cycle-aligned day ranges, steroid taper periods). Temporal anchoring reduces false positives from historical or duplicate statements and clarifies whether the toxicity is active. Pharmacist review then confirms clinical relevance before any order change is considered.

### 5.5. Keep Pharmacists in the Loop by Design

A pharmacist-in-the-loop workflow should be explicit and embedded in routine order verification, not treated as an optional safety check. Model outputs should enter a dedicated pharmacist review queue within the EHR order-verification workspace, where each item presents a structured summary of the suspected AE episode (drug/regimen, CTCAE term and grade, temporal window, and supporting evidence spans) and any candidate actions (hold/reduce/delay/rechallenge/monitor). Pharmacists review these items and can accept, modify, or dismiss suggestions, with high-risk scenarios (e.g., grade ≥ 3 toxicities or narrow-therapeutic-index agents) always requiring pharmacist confirmation before any order change.

Each decision should be accompanied by concise reason codes (e.g., not active, wrong grade, not drug-related, insufficient evidence, conflicts with local protocol). This preserves a human safety gate, generates supervision signals for iterative model improvement, and creates an auditable link between model outputs and order-level decisions. Mapping reason codes to a compact error taxonomy (wrong fact, misapplied rule, temporal error, hallucination) enables targeted updates and governance. In this design, the model compiles and structures the evidence, but the pharmacist remains the operating control and final sign-off authority for therapy decisions.

### 5.6. Tune Precision to the Intended Use

Operating points shape workload and trust. High recall with modest PPV is appropriate for surveillance queues, whereas “ready-to-recommend” suggestions require higher precision. Thresholds should be co-determined with pharmacists and periodically recalibrated using acceptance/override rates and observed error types, with thresholds tailored by AE category (e.g., stricter for high-risk toxicities) to align model behavior with the intended role in order verification.

### 5.7. Measure Decision Impact, Not Detection Alone

Prospective evaluations should report endpoints that reflect decision quality and safety: grade-to-action correctness against a specified CTCAE version or local policy; prevented harms (e.g., averted grade ≥ 3 progression before administration); and operational metrics (pharmacist time per case, alert burden, acceptance/override, secondary review requests). These measures demonstrate improvement beyond detection accuracy and are essential for practice adoption.

### 5.8. Standardize Versioning and Error Taxonomies

Reproducibility requires consistent reporting and version pinning (CTCAE, prompts, model weights, retrieval corpora). Implementations should specify the CTCAE version and any guideline/label versions/dates, describe the reference-standard process (e.g., expert adjudication procedures and agreement), and classify unsafe recommendations with a compact taxonomy (wrong fact, misapplied rule, temporal error, hallucination). Standardization enables meaningful comparison, governance, and audit.

### 5.9. Build Pharmacist-Centric Benchmarks

To align development with pharmacy work, curate de-identified benchmarks containing CTCAE-labeled spans, patient-level AE episodes with temporal boundaries, and action labels (hold/reduce/delay/rechallenge/monitor), stratified by note type. Such datasets allow evaluation of what ultimately determines order-level decisions, not just detection or grade.

### 5.10. Integrate and Govern Before You Automate

Delivering outputs into the pharmacist’s EHR order-verification workspace as structured artifacts (e.g., flowsheet rows or AE entries) with side-by-side evidence. Governance should include version pinning for CTCAE and local guidance, change logs for prompts/models/sources, and fail-safe defaults (no action suggested when confidence or provenance is inadequate). Rollouts should progress from silent mode to assisted review, and only then to limited candidate-action pilots, always retaining pharmacist sign-off.

### 5.11. Test Generalizability and Equity

Validate systems across institutions, note styles, and populations (including non-English notes where relevant), with routine monitoring for performance disparities by age, sex, race/ethnicity, language, regimen, and care setting. Pharmacist feedback should guide targeted adaptation when subgroup gaps are detected. The objective is not only high average performance but reliability across patient groups and care settings, consistent with the pharmacist-centered goals defined in the Introduction.

## 6. Conclusions

This review of 19 empirical studies demonstrates that AI on text data has the potential for adverse-event surveillance in oncology and can help to reduce the effort required to surface clinically relevant signals from narrative notes. Detection is the most mature capability; CTCAE grading is achievable when the task is constrained to structured outputs and temporally anchored. These findings align closely with contemporary oncology pharmacy workflow: AI compiles the evidence, and the pharmacist verifies and determines the course of action before any order change. Thus, for practice, the safest and most impactful role for LLM-based systems is as pharmacist-in-the-loop tools that surface candidate AEs, provide CTCAE-aligned summaries with citations, and support triage rather than autonomous order changes. The field now needs prospective and multi-center evaluations that report grade-to-action correctness, prevented harms, time-per-case, and pharmacist acceptance/override, along with consistent CTCAE versioning and transparent governance. With these guardrails, LLMs can help oncology pharmacists act faster and more consistently, while keeping therapy decisions under human control.

## Figures and Tables

**Table 1:** Glossary of terms and acronyms with full meaning.

Term	Definition/Full Meaning	Term	Definition/Full Meaning
ADC	Antibody–drug conjugate	ADE	adverse drug event
AE(s)	Adverse events	AI	artificial intelligence
ARAT	androgen receptor-axis-targeted agents	ASCO	American Society of Clinical Oncology
ASTCT	American Society for Transplantation and Cellular Therapy	AUPRC	area under the precision–recall curve
BC	breast cancer	BERT	Bidirectional Encoder Representations from Transformers
BERTopic	topic modeling algorithm	BWH	Brigham and Women’s Hospital
CAR-T	chimeric antigen receptor T-cell	CI	confidence interval
Cox	Cox proportional hazards (model)	CPOE	Computerized provider order entry
CRPC	castration-resistant prostate cancer	CRS	Cytokine release syndrome
CTCAE	Common Terminology Criteria for Adverse Events	ECOV	Electronic chemotherapy order verification
EHR	Electronic health record	eMAR	Electronic medication administration record
F1 (F-measure)	harmonic mean of precision and recall	FDA	Food and Drug Administration
FN	false negative	FP	false positive
GPT	generative pre-trained transformer	GPT-4	Generative Pre-trained Transformer 4
HER2+	human epidermal growth factor receptor 2-positive	HFS	hand-foot syndrome
HIPAA	Health Insurance Portability and Accountability Act	HOPA	Hematology/Oncology Pharmacy Association
HR	hazard ratio	ICANS	Immune effector cell–associated neurotoxicity syndrome
ICD	International Classification of Diseases	ICD-10	International Classification of Diseases, 10th Revision
ICI	Immune checkpoint inhibitor	ILD	interstitial lung disease
IR	interventional radiology	irAE	Immune-related adverse events
JSON	JavaScript Object Notation	Kendall W	Kendall’s coefficient of concordance
LLM(s)	Large language models	LLT	Low Level Term
MedDRA	Medical Dictionary for Regulatory Activities	medspaCy	medical spaCy (clinical NLP toolkit)
MEPS	myocarditis, encephalitis, pneumonitis, and severe cutaneous adverse reactions	MGH	Massachusetts General Hospital
MoA	mechanism of action	MWA	microwave ablation
NCCN	National Comprehensive Cancer Network	NER	named entity recognition
NLP	Natural language processing	NPV	negative predictive value (mentioned in linked studies; if you cite them later in Results, include here)
OICI	Osaka International Cancer Institute	OncoBERT	oncology-domain BERT model
ONS	Oncology Nursing Society	*p*	*p*-value
PPV	Positive Predictive Value	Proximal management signal	an indirect marker of AE management (e.g., steroid initiation) when direct grade-to-action measurement isn’t available
PSM	propensity score matching	pt/pts	patients
PT	Preferred Term(s)	PubMedBERT	BERT pretrained on PubMed biomedical text
Q&A	question and answer	R11	nausea and vomiting
R53	malaise and fatigue	RAG	Retrieval-augmented generation
RT	radiotherapy	RW	real-world
RWE	Real-world evidence	SCAR	severe cutaneous adverse reaction
SITC	Society for Immunotherapy of Cancer	SOAP	Subjective, Objective, Assessment, Plan
T-DM1	trastuzumab emtansine	T-DXd	trastuzumab deruxtecan
TI/AB	Title/Abstract (database search fields)	TN	true negative
TP	true positive	UCSF	University of California, San Francisco
Version pinning	fixing versions of CTCAE, prompts, model weights, and retrieval sources to ensure traceability and reproducibility	VUMC	Vanderbilt University Medical Center

**Table 2:** Summary of 19 articles. For the acronyms, please see Table 1.

Study Objective	Clinical Data Source and Size	LLM Approach	Metric and Performance	Clinical Impact/Advancements	Author and Year
Use Llama-3.1 to detect five therapy-related AEs from clinical notes and predict time-to-AE, validated against prospectively collected trial AE data.	Prospective AE gold standard from 1754 solid-tumor pts across 675 trials; note-level subset of 100 curated notes.	Llama 3.1 LLM AE annotation; CTCAE v5.0 definitions; note- and patient-level evaluation; Pearson R^2^ for time-to-AE.	Adrenal insuff: note sens/spec 100/97.8; patient 97.7/94.7; R^2^ 98.2. Colitis: 66.7/99.0; 94.3/80.4; 89.2. Hyperthyroid: 57.1/100; 74.0/91.4; 98.7. Hypothyroid: 100/88.9; 88.1/74.0; 96.1. Pneumonitis: 76.9/97.7; 98.6/70.1; 83.9.	Demonstrates accurate, scalable LLM-based AE surveillance across cancers; enables translational toxicity research using routinely documented clinical text.	Bakouny et al., 2025 [34]
Identify ICI-induced irAEs from EHR text using LLM and assess patient impact (steroids, discontinuation) versus diagnosis codes.	Mayo Clinic EHR (2005–2021): unstructured notes + structured orders/codes; 9290 ICI-treated adults; manual-review MEPS cohort; comparators: diagnosis codes, medication orders.	Augmented curation using fine-tuned SciBERT sentence classifier (drug-to-phenotype) plus medication-administered classifier; synonym libraries; ensemble over phrase fragments to infer ICI → irAE causality and steroids.	Drug-to-phenotype acc 84.8%, precision/recall >85%; medication-administered acc 89%, precision/recall >88%; MEPS vs. manual: spec 0.858, sens 0.903, PPV 0.792, NPV 0.937, F1 0.844; steroid use 82%.	Outperformed diagnosis codes for causal irAE detection; quantified severity actions (steroids, discontinuation); enables scalable real-world pharmacovigilance and safety profiling for ICIs.	Barman et al., 2024 [35]
Assess GPT-3.5/4/4o for identifying immune-related adverse events from EHR notes and clinical trial narratives across institutions.	VUMC EHR (100 pts; 26,432 notes), UCSF EHR (70 pts; 487 notes), 7 Roche trials (272 pts; narratives); comparator: manual irAE annotations/MedDRA database.	Zero-shot GPT-3.5/4/4o (Azure OpenAI); prompt with irAE lists/synsets; JSON outputs; patient-level aggregation thresholds; organ-level mapping.	Patient-level micro-F1: VUMC 0.556–0.589; UCSF 0.581–0.662; Roche 0.535–0.620. Note-level (VUMC) irAE micro-F1 0.496–0.571; category micro-F1 0.611–0.656.	Generalizable LLM irAE phenotyping across EHRs and trials; scalable adverse-event surveillance from text; reduces manual review burden and supports pharmacoepidemiologic safety monitoring.	Bejan et al., 2025 [36]
Evaluate chatbot accuracy/completeness for irAE management across organ systems and scenarios.	50 guideline-derived questions (10 irAE categories) + 20 patient-specific scenarios; 8 expert raters; comparator: ASCO/SITC/NCCN guidelines.	ChatGPT (GPT-4) and Google Bard; zero-shot Q&A; expert Likert ratings (1–4) for accuracy/completeness.	ChatGPT: mean accuracy 3.87, completeness 3.83; Bard: 3.50, 3.46; *p* < 0.001 overall. Ratings of 1: 0.3% vs. 1.1%. Scenarios: accuracy 3.73, completeness 3.61; Kendall W ~0.21–0.27.	Shows GPT-4 provides largely accurate, guideline-consistent irAE management guidance; supports clinician use with verification and highlights category-specific gaps.	Burnette et al., 2024 [37]
Evaluate GPT-4 for classifying chemotherapy-induced subjective toxicities vs. oncologists using CTCAE v5.	30 fictitious audio cases (transcribed); 13 oncologists; comparator: oncologist mode/mean CTCAE grades.	Contextualized GPT-4 with CTCAE v5 reference; custom GPT; 10 runs/case; outputs general (none/mild/severe) and specific grades 0–4.	General-category accuracy 81.5–85.7%; specific-category accuracy 64.4–64.6%; mild errors 96%, severe errors 3.6–4%; false alarms 3–8.9%.	Demonstrates near-expert general severity classification from narratives; feasible LLM toxicity monitoring; needs improvement for fine-grained grading; basis for real-patient validation.	Ruiz Sarrias et al., 2024 [38]
Classify CTCAE esophagitis severity from radiotherapy clinic notes.	Gold: 1524 notes/124 lung pts; Silver: 2420 notes/1832 pts; External: 345 notes/75 esophageal pts; comparator: manual CTCAE labels & structured toxicity grades.	Encoder-only PubMedBERT fine-tuned; medspaCy sectionizer; CTCAE v5.0 mapping; silver-label augmentation; note → patient aggregation.	Note macro-F1: 0.92 (Task1), 0.82 (Task2), 0.74 (Task3); external 0.73/0.74/0.65. Patient macro-F1: 1.00/0.92/0.49 (lung) and 0.70/0.69/0.58 (esophageal).	First to automatically extract CTCAE severity from notes; supplements sparse ICD-10 coding; enables scalable radiotherapy (RT) toxicity surveillance and RWE.	Chen et al., 2023 [39]
Use BERT-based NLP plus clinical review to identify ADC-related AEs in HER2+ breast cancer.	Mayo Clinic EHR notes; T-DXd *n* = 266, T-DM1 *n* = 432; manual adjudication for ILD/colitis causality.	Fine-tuned BERT classifier on time-stamped notes with synonym lists; clinician adjudication of drug → AE causality.	Adjudicated ILD = 16 (14 T-DXd, 2 T-DM1); steroids 15/16 (94%); hospitalized 9/16 (56%); fatalities 3/16 (19%); colitis 0 attributable.	Demonstrates scalable AE surveillance from EHR text for ADCs; quantifies real-world ILD burden and management outcomes to support pharmacovigilance.	Chumsri et al., 2025 [40]
Assess GPT-3.5 feasibility for extracting post-ablation complications and local recurrence from oncology reports/notes.	Single-center EHR: 20 lung MWA patients; 104 radiology reports + 37 clinic notes; comparator: manual chart-review ground truth.	GPT-3.5 Turbo 16 k (Azure HIPAA) zero-shot; temperature 0; prompt for four binary outcomes (recurrence, pneumothorax, hemoptysis, hemothorax); Python pipeline.	Recurrence: acc 85%, recall 100%, precision 77%, F1 0.87. Pneumothorax: acc 85%, recall 67%, precision 100%, F1 0.80. Hemoptysis acc 95% (1 FP). Hemothorax acc 100%.	Demonstrates LLM-based extraction of oncology IR outcome AEs from routine text; supports faster registry curation and safety surveillance.	Geevarghese et al., 2025 [41]
Automate extraction of late RT-related toxicities from prostate cancer clinical notes using an LLM.	Single-center EHR; 177 pts; 1133 notes (>6 mo post-RT); 699 notes for optimization; 434-note validation; 12 GU/GI domains.	Teacher–student: Mixtral-8x7B extracts symptoms; GPT-4 refines prompts over 16 rounds/5 epochs; outputs positive/negative/neutral with rationale.	Single-symptom notes: acc 0.71, precision 0.82, recall 0.71, F1 0.73; multi-symptom best acc: hematuria 0.76, UC 0.70; per-symptom postrefinement acc 72–97%, overall ~84%.	Shows feasible LLM-based surveillance of late RT toxicities; iterative teacher–student refinement improves extraction; supports longitudinal toxicity monitoring in prostate cancer.	Ghanem et al., 2025 [42]
Evaluate GPT-4 for extracting CAR-T adverse events and building AE timelines from progress notes.	UCSF deidentified EHR; 4183 notes within 30 days post-CAR-T from 253 patients; ~10% (25 pts) manually validated by clinician.	GPT-4 (Azure HIPAA) zero-shot AE extraction for events prompting clinical intervention; BERTopic clustering to identify temporal patterns.	Manual validation accuracy 64%; 19 AE clusters; largest cluster (hyponatremia/leukocytosis/encephalopathy/neurologic) occurred mean 12.9 days post-CAR-T.	Shows feasibility of LLM-based AE surveillance and timeline construction after CAR-T, potentially reducing manual chart-review burden.	Guillot et al., 2024 [43]
Assess domain adaptation for ADE extraction from breast cancer EHR notes.	OICI pharmacy notes (Japanese): 1928 notes from 434 breast cancer patients (2019–2021); 1000 notes annotated; plus 1027 dummy case reports; comparator: held-out annotated test set.	Encoder-only BERT (cl-tohoku/bert-base-japanese-char-v2) fine-tuned for NER; domain adaptation with 100/500/800 in-domain notes; MedDRA normalization via Levenshtein matching.	Baseline F1 = 0.51; +40% F1 with 100 notes; best with 800 notes F1 = 0.84; 10,569 mentions normalized to 343 MedDRA PTs; regimen-wise ADE frequencies tabulated.	Demonstrates LLM-based ADE detection in Japanese EHRs; domain adaptation markedly improves performance; enables regimen-specific ADE surveillance to support pharmacovigilance.	Andrade et al., 2024 [44]
Compare GPT-4 oncology drug safety information with FDA labels.	53 solid-tumor drugs with 2020–2022 approvals; comparator: FDA package inserts; two-physician review.	GPT-4 Q&A for four items; safety items: common AEs ≥20% and warnings/precautions; repeat-query check for discordances.	Indications/MoA acc = 100%; AEs correct = 53%, incorrect = 47% warnings/precautions correct = 32%, incorrect = 68% (36/53); repeated queries changed AE answers 76% and warnings 53%.	Reveals omissions/variability in GPT-4 safety outputs; cautions against relying on GPT as a primary oncology drug safety source.	Hundal et al., 2024 [45]
Detect capecitabine-induced HFS from EHR notes with BERT-based NER and evaluate celecoxib’s preventive effect.	University of Tokyo Hospital EHR (2004–2021): 44,502 cancer pts; 669 capecitabine users; PSM 636 vs. 636; celecoxib 31 vs. 31; manual validation 2606 notes/62 pts; comparator: manual annotations.	MedNERN-CR-JA (Japanese BERT) NER with ADR normalization; positive-attribution filtering; patient-level aggregation; propensity score matching; Cox models for HFS risk.	Patient-level: precision 0.875, recall 1.000, F1 0.933; document-level: precision 0.920, recall 0.857, F1 0.888; capecitabine vs. none HR 1.93 (95% CI 1.48–2.52); celecoxib HR 0.51 (0.24–1.07).	Demonstrates reliable LLM-based detection of symptomatic AE (HFS) from real-world Japanese EHR and enables retrospective safety evaluation suggesting celecoxib’s protective trend.	Tsuchiya et al., 2025 [46]
Detect symptomatic AEs from oncology notes and estimate drug-associated time-to-event risk.	University of Tokyo Hospital EHR (progress, discharge, nursing notes) 2004–2021; *N* = 39,319 cancer patients; PSM comparator cohorts.	MedNERN-CR-JA (Japanese BERT) NER with AE dictionary; onset = first symptom after drug; 1:1 PSM; Cox models.	Significant HRs: anthracyclines → cardiotoxicity 1.21; oxaliplatin → peripheral neuropathy 2.56; capecitabine → HFS 1.93; detectability varies by note source (Figure 2).	Validates large-scale LLM-based AE surveillance and time-to-onset analysis from notes, informing pharmacovigilance beyond codes/labs and emphasizing data-source choice.	Tsuchiya et al., 2025 [47]
Predict nausea/vomiting and fatigue/malaise events from oncology clinical notes.	Centre Léon Bérard EHR (France); 140,523 patients; 2,515,957 notes; labels: ICD-10 events within 90 days of notes.	OncoBERT (BERT, French) pretrained on local notes; time-encoding of sequential reports; fine-tuned vs. DrBert/K-memBERT; random undersampling.	Macro-AUPRC: 0.58 (OncoBERT) vs. 0.50 (open-source baseline) for R11/R53 prediction.	Demonstrates large-scale LLM prediction of common oncologic AEs from notes to enable early alerts and reduce AE-related hospitalizations.	Vienne et al., 2024 [48]
Identify adverse events from community-pharmacy records of ARAT-treated prostate cancer outpatients using BERT NER.	Nakajima Pharmacy Group records (Japan, 2020–2021); Step1: 1008 annotated notes; Step2: 161 ARAT patients, 2193 assessment notes; comparator: manual annotation.	MedNER-CR-JA (Japanese BERT) NER with factuality tags (positive/suspicious/negative/general); preprocessing; Fleiss κ = 0.62; evaluation by exact/position matches.	Exact-match F1 = 0.72 (positive 0.70; negative 0.78); position-match F1 = 0.86; 1900 symptom tags from 2193 notes (32.8% positive).	Enables scalable AE surveillance from community-pharmacy narratives, capturing drug-specific ARAT AE profiles and documenting absence of severe AEs for outpatient safety monitoring.	Yanagisawa et al., 2025 [49]
Test LLM generalizability for drug–ADE relation extraction across datasets.	The Ohio State University (OSU) ICI notes: 1394 notes/47 pts; 189 relations. n2c2: 505 summaries; 1355 relations; gold standards = manual annotations.	Encoder LLMs (BERT, ClinicalBERT) on sentence text between drug and ADE; compared vs. SVM/CNN/BiLSTM.	ClinicalBERT F-score inter-dataset: 0.78 (OSU → n2c2), 0.74 (n2c2 → OSU); intra-dataset best on n2c2: 0.87.	Shows superior cross-site ADE detection from notes with ClinicalBERT, supporting scalable oncology pharmacovigilance.	Zitu et al., 2023 [50]
Compare NLP/LLM Augmented Curation vs. ICD/manual review for detecting ICI irAEs and associated actions.	Mayo Clinic EHR; ~9000 ICI-treated pts; gold-standard manual A.C.H. cohort; 540 patients with A.C.H.; comparator: ICD codes.	SciBERT-based Augmented Curation with sentence extraction/entity classification for drug–AE causality; assesses steroid/2 L immunosuppressant use and ICI discontinuation; time-to-curate benchmark.	F1 = 0.85 for drug–AE; higher sensitivity/PPV/NPV than ICD; processing ~10 min for 9000 pts vs. ~9 weeks manual.	Quantifies irAE management actions (steroids 79%, 2 L immunosuppressant 6.1%, ICI discontinuation 7.7%); demonstrates scalable, accurate safety surveillance from unstructured notes.	Block et al., 2023 [51]
Evaluate an LLM vs. ICD codes for detecting severe ICI-related irAEs from EHR text.	MGH inpatient EHR: 7555 admissions (2011–2023); external validation BWH: 1270 admissions (2018–2019); comparator: manual adjudication and ICD codes.	Open-source Mistral OpenOrca with retrieval-augmented generation; four irAE prompts; vector database; zero-shot yes/no outputs; ~9.53 s/chart.	MGH: mean sens 94.7%, spec 93.7%, PPV 15.2%, NPV 99.9%; significant gains vs. ICD for hepatitis, myocarditis, pneumonitis. Validation: sens 98.1%, spec 95.7%; adjusted sens/spec 99.2%/97.6% (model-detected).	Outperforms ICD coding and accelerates adjudication; generalizes across institutions; identifies missed irAEs, enabling scalable safety surveillance.	Sun et al., 2024 [52]

## Data Availability

No new data were created or analyzed in this study. Data sharing is not applicable to this article.
